# Osmotic Adaptation and Compatible Solute Biosynthesis of Phototrophic Bacteria as Revealed from Genome Analyses

**DOI:** 10.3390/microorganisms9010046

**Published:** 2020-12-26

**Authors:** Johannes F. Imhoff, Tanja Rahn, Sven Künzel, Alexander Keller, Sven C. Neulinger

**Affiliations:** 1 GEOMAR Helmholtz Centre for Ocean Research, 24105 Kiel, Germany; trahn@geomar.de; 2 Max Planck Institute for Evolutionary Biology, 24306 Plön, Germany; kuenzel@evolbio.mpg.de; 3 Center for Computational and Theoretical Biology, University Würzburg, 97074 Würzburg, Germany; a.keller@biozentrum.uni-wuerzburg.de; 4 omics2view.consulting GbR, 24118 Kiel, Germany; s.neulinger@omics2view.consulting

**Keywords:** genomes of photosynthetic bacteria, glycine betaine biosynthesis, ectoine biosynthesis, osmotic adaptation, phylogeny of osmolyte biosynthesis

## Abstract

Osmotic adaptation and accumulation of compatible solutes is a key process for life at high osmotic pressure and elevated salt concentrations. Most important solutes that can protect cell structures and metabolic processes at high salt concentrations are glycine betaine and ectoine. The genome analysis of more than 130 phototrophic bacteria shows that biosynthesis of glycine betaine is common among marine and halophilic phototrophic *Proteobacteria* and their chemotrophic relatives, as well as in representatives of *Pirellulaceae* and *Actinobacteria*, but are also found in halophilic *Cyanobacteria* and *Chloroherpeton thalassium.* This ability correlates well with the successful toleration of extreme salt concentrations. Freshwater bacteria in general lack the possibilities to synthesize and often also to take up these compounds. The biosynthesis of ectoine is found in the phylogenetic lines of phototrophic *Alpha*- and *Gammaproteobacteria*, most prominent in the *Halorhodospira* species and a number of *Rhodobacteraceae*. It is also common among *Streptomycetes* and *Bacilli*. The phylogeny of glycine-sarcosine methyltransferase (GMT) and diaminobutyrate-pyruvate aminotransferase (EctB) sequences correlate well with otherwise established phylogenetic groups. Most significantly, GMT sequences of cyanobacteria form two major phylogenetic branches and the branch of *Halorhodospira* species is distinct from all other *Ectothiorhodospiraceae*. A variety of transport systems for osmolytes are present in the studied bacteria.

## 1. Introduction

Phototrophic bacteria are widely distributed at suitable habitats in the marine and hypersaline environment. They are exposed to sometimes dramatically changing salt concentrations and some are found in saturated brines of salt and soda lakes, where they regularly develop massive blooms, often forming patches and pinkish-red layers, even within deposits of crystalized salts [1,2]. One of the prerequisites to cope with high salt and solute concentrations is the ability to keep an osmotic balance, i.e., a positive turgor pressure inside the cells through the accumulation of solutes in the cytoplasm that are compatible with the metabolic processes, even at high concentrations, and preserve active structures of proteins and nucleic acids [3]. In consequence, these bacteria need proper mechanisms of osmotic adaptation and ways to accumulate osmotically active compatible solutes up to several molar concentrations at the extremes.

Limitation to protect cell structures and metabolism is given by the compatibility of the solutes and the ability to accumulate to high or extremely high, several molar concentrations inside the cell. Glycine betaine (in some cyanobacteria also glutamate betaine) and ectoine (also hydroxyectoine) are the top candidates for this function in bacteria. Glycine betaine (hereafter “betaine”) accumulation is widespread among phototrophic and chemotrophic eubacteria [4,5,6,7,8]. Ectoine was first identified as a compatible solute in the extreme halophilic *Halorhodospira halochloris* [9] and was later shown to be widely distributed among marine and halophilic eubacteria [10]. Though a number of other solutes are accumulated in bacteria in response to osmotic stress, these can provide protection only at low to moderate osmotic stress. Such compounds include sugars such as trehalose and sucrose, amino acids in particular glutamate, glucosyl glycerol, N-acetyl-glutaminyl glutamine amide, N-carbamoyl-glutamine amide, and others [6,7,11,12,13]. Basically, the accumulation can be achieved by uptake from the environment or by biosynthesis.

Betaine biosynthesis can be achieved by three consecutive methylation steps from glycine and includes the formation of monomethylglycine (sarcosine) and dimethylglycine as intermediates. In most of the bacteria studied, these enzymes are encoded by two genes and have overlapping enzymatic activities. In *Halorhodospira halochloris*, the first enzyme (glycine and sarcosine methyltransferase GMT) catalyzes the formation of monomethylglycine and dimethylglycine and the second one (dimethylglycine methyltransferase DMT) catalyzes the methylations to dimethylglycine and betaine [14,15]. In the halophilic cyanobacterium *Aphanothece halophytica*, the second enzyme specifically catalyzes the methylation of dimethylglycine [16]. In *Actinopolyspora halophila*, the two genes are fused, showing corresponding sequence homologies to the two genes in *Halorhodospira halochloris* [14].

An alternative route of betaine biosynthesis starts from choline and oxidizes this compound in two steps to betaine, catalyzed by choline dehydrogenase (BetA) and betaine aldehyde dehydrogenase (BetB). This route is widely distributed among bacteria but requires the external presence and uptake of choline. One possible uptake system is the high-affinity secondary transporter BetT, considered to be a specific choline transporter in *E. coli* [17] and a betaine transporter in *Aphanothece halophytica* [18]. In *E. coli*, the *betT* gene, together with a regulatory *betI* gene, is included in the *bet* gene cluster.

The biosynthesis of ectoine, which was first identified in the extreme halophilic phototrophic bacterium *Halorhodospira halochloris* [9], starts from aspartate. Aspartate is activated to L-aspartate-phosphate (Ask_ect) and then reduced to L-aspartate-β-semialdehyde (Asd), followed by a transamination (with glutamate or alanine as donor of the amino group) to L-diaminobutyric acid (EctB), acetylation of the amino group to N-acetyldiaminobutyric acid (EctA), and finally ring closure (EctC) to form ectoine, as shown for *Halorhodospira halophila* and *Halomonas elongata* [19,20]. Genes responsible for ectoine biosynthesis and their osmotically regulated expression were first identified in *Marinococcus halophilus* [21]. The oxidation of ectoine to hydroxyectoine, which was first demonstrated in *Streptomyces parvulus* [22], turned out to be common to many Actinobacteria. The hydroxylation of ectoine (EctD) is strongly dependent on the presence of molecular oxygen and is accompanied by the oxidative decarboxylation of oxoglutarate forming CO_2_ and succinate [23]. While the *ask_ect* gene, which encodes a specific aspartate kinase not underlying the feedback control of threonine [24,25], is often included in the *ect* gene cluster, *asd* usually is at a different locus in the genome. The coexpression of *ask_ect* together with the osmotically induced gene cluster *ectABC*, ensures optimal supply of the precursor L-aspartate-β-semialdehyde under osmotic stress conditions. Aspects of ectoine and hydroxyectoine biosynthesis were recently reviewed by Czech et al. [10].

Osmotic adaptation can also be achieved by uptake of osmolytes rather than biosynthesis. Provided that such solutes are available in the environment, uptake generally is the favored way, because it is far less energetically expensive than de novo synthesis. Laboratory culture media with complex carbon sources (e.g., proteose peptone and yeast extract), often contain these compounds. In such media, betaine was accumulated to high levels exceeding 1 M concentrations by a number of salt-tolerant chemoheterotrophic bacteria isolated from hypersaline soils [5]. Additionally, a number of phototrophic green and purple sulfur bacteria are able to take up and accumulate betaine but are incapable of its biosynthesis [13]. In nature, such situations can occur, whenever large decaying biomass is accumulated and betaine or choline becomes available, leaking from living cells or released upon cell lysis. Under such conditions, which are likely found in microbial mats, uptake might become an important strategy to accumulate compatible solutes, although dependent on biosynthesis as their primary producers. Uptake systems for betaine, choline, and ectoine are found in numerous bacteria, many of which are unable to biosynthesize these molecules. Best known is a widely distributed transport system first identified in the transport of proline ProU (ProVWX), which has low affinity to choline and also transports glycine betaine, proline betaine, carnitine, and ectoine [26,27]. Another betaine transport system is OpuA. Both of these systems are ABC type transport systems and include binding proteins for the substrate and for ATP and a permease (ProVWX, OpuAA,AB,AC). Several more other transport systems for osmolytes are known (see [27]). Of these, OpuD is a single-component secondary transporter for betaine. BetT might also be active in betaine transport as found in *Aphanothece halophytica* [18].

In the present study, we focused on the biosynthetic capability of phototrophic bacteria to produce betaine and ectoine, using genomic information available from new genome sequences and from databases. The genomic repertoire and the distribution of the studied biosynthesis pathways are related to salt responses of the bacteria to conclude on their requirements for environmental adaptation. In addition, the phylogeny of ectoine and betaine biosynthesis was studied by comparing sequences of glycine–sarcosine–methyltransferase (GMT) and diaminobutyrate–pyruvate aminotransferase (EctB) of phototrophic bacteria, together with selected chemotrophic bacteria.

## 2. Materials and Methods

### 2.1. Cultivation and DNA Extraction

Cells were grown in the appropriate media, as described for the purple sulfur bacteria [28,29] and several groups of phototrophic nonsulfur purple bacteria [30]. Extraction of DNA was done as described earlier [31]. DNA from 2 mL of a freshly grown culture was extracted with the DNeasy^®^ Blood&Tissue Kit, according to the manufacturer’s instructions (QIAgen, Hilden, Germany), including the pretreatment for Gram-positive bacteria (consisting of enzymatic lysis buffer, proteinase K, and RNAse) and then dissolved in the TE-buffer. The extracted DNA was checked for quantity and quality by agarose gel electrophoresis with linear and double-stranded Lambda DNA used as control (Thermo Fisher Scientific, Waltham, MA, USA, Cat.No. SD0011). DNA was purified using a gel extraction procedure with the MoBio Ultra DNA Purification Kit (Cat.No. 12100-300).

### 2.2. Sequencing and Assembly

Sequencing of DNA and the assembly of sequences were done, as described earlier [31]. Samples were prepared with the Nextera^®^ XT DNA Sample Preparation kit from Illumina, following the manufacturer’s protocol. Afterwards, the samples were pooled and sequenced on the MiSeq using the MiSeq^®^ Reagent Kit v3 600 cycles sequencing chemistry. The library was clustered to a density of approximately 1200 K/mm^2^.

Read quality filtering was performed with Trimmomatic v0.36 [32]. Reads were scanned for residues of Illumina Nextera XT adapters. Quality trimming was conducted with a 5-base pairs (bp) sliding window, trimming the read once the average Phred quality score within this window dropped below 30. Reads with a minimum length of 21 bp after quality trimming were retained. Single reads (i.e., reads with their mate deleted) were retained and included into downstream analysis. Reads were further checked for ambiguous base calls as well as for low complexity, employing the DUST algorithm [33]. They were filtered accordingly with an in-house R script in Microsoft R Open v3.3.2 (R Core Team 2016). Retained reads are referred to as ‘filtered reads’. Filtered reads were pre-assembled with SPAdes v3.10.0 [34,35], using default k-mer lengths. Scaffolds ≥500 bp of this pre-assembly were subjected to extension and second-round scaffolding with SSPACE standard v3.0 [36].

### 2.3. Genome Annotation and Submission to GenBank

Genome sequences were annotated by the “Rapid Annotation using Subsystem Technology” (RAST) [37]. Sequences of EctB, GMT, and ProW were retrieved from the annotated genomes using the RAST and The SEED Viewer provided by this platform [38,39], which also offered the option to search with gene/protein sequences within the annotated genomes. In addition, standard protein BLAST of the NCBI database was used with EctB and GMT sequences to retrieve additional protein sequences. All genome sequences were deposited in the GenBank database (Supplementary Table S4). Accession numbers of gene and genome sequences, together with species and strain designations as well as the corresponding higher taxonomic ranks, are included in Supplementary Tables S1–S4.

### 2.4. Phylogenetic Sequence Analyses

For phylogenetic analysis, protein sequences of GMT, EctB, and ProW were aligned using ClustalX version 2.1 [40] and the trees were calculated by the neighbor-joining (NJ) method with correction for multiple substitutions, according to ClustalX [41]. NJ plot was used to draw the phylogenetic trees expressed in the Newick phylogenetic tree format [42]. The tree topologies were evaluated with bootstrap analyses, based on 1000 replicates and the values are indicated in the trees.

## 3. Results and Discussion

### 3.1. Osmotic Adaptation in Phototrophic Bacteria

According to genome analysis of representative phototrophic bacteria, the genetic repertoire and kind of possible responses to osmotic stress of these bacteria varies widely between the different groups and between freshwater, marine, and halophilic species. The genetic repertoire of all studied phototrophic bacteria is shown in Table 1, Table 2 and Table 3. While almost all marine and halophilic phototrophic bacteria can synthesize either betaine or ectoine or both, true freshwater bacteria lack the ability to synthesize betaine and ectoine. Often, they also lack the possibility of uptake of these osmolytes or their biosynthetic precursors, while marine and halophilic bacteria generally have this option. Obviously, biosynthesis or uptake of betaine more than that of ectoine is a prerequisite for their ability to thrive in marine and hypersaline habitats and to tolerate high salt concentrations.

The ability to produce betaine or ectoine from the currently known routes is absent from the freshwater bacteria examined here, which include *Heliobacteria*, *Chloracidobacterium*, *Chloroflexi*, and the majority of *Cyanobacteria* and *Chlorobiaceae*, as well as phototrophic *Betaproteobacteria* (Table 1). Most of these bacteria also lack corresponding transport systems. Both pathways are also absent from the phylogenetic groups of freshwater phototrophic species of *Rhizobiales*, *Acetobacteraceae*, *Rhodospirillaceae*, *Betaproteobacteria*, and *Chromatiaceae* (Table 1, Table 2 and Table 3). In all freshwater bacteria that lack any of the options to accumulate betaine or ectoine, a limited osmotic adaptation might be achieved by accumulation of sugars, in particular trehalose and sucrose or glucosylglycerol, but also N-acetyl-glutaminylglutamine amide and N-carbamoyl-L-glutamine amide (Severin et al., 1992). Even potassium glutamate to some extent might contribute to osmotic adaptation [1,6,7].

#### 3.1.1. Cyanobacteria

It was demonstrated that the salt-tolerance of *Cyanobacteria* was clearly related to the compatible solutes accumulated, those of the lowest tolerance (freshwater strains growing below 0.7 M NaCl) accumulate sucrose and trehalose, those of moderate tolerance (marine strains growing up to 1.8 M NaCl) accumulate glucosylglycerol, and those with the highest tolerance (strains of marine and hypersaline origin, in great majority classified as *Synechococcus* strains) accumulate glycine betaine or glutamate betaine [11]. Apparently, the majority of cyanobacteria adapted to marine environments count on glucosylglycerol as osmoticum [43,44,45]. The formation of a fused molecule with glycerol (glucosyl glycerol) as a component appears to be a clever strategy to keep at least, in part, the excellent compatible nature of glycerol but reduce leakage through the cell membrane. Those *Cyanobacteria* originating from salt lakes and hypersaline ponds (tolerance of >20% NaCl) accumulate betaine, which apparently is essential to provide sufficient protection at moderately and extremely high salt concentrations [11].

**Figure 1:**
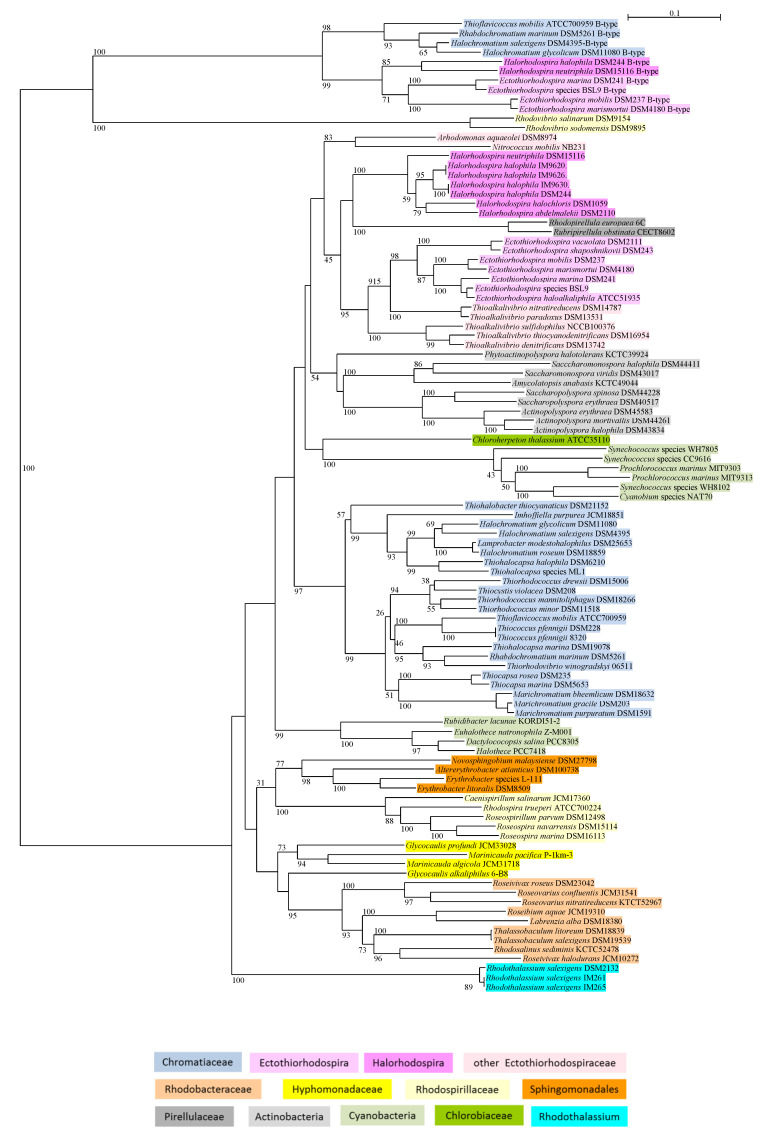
The phylogeny of betaine biosynthesis based on the sequences of glycine/sarcosine methyltransferase GMT is shown in a neighbor-joining tree. Sequences and gene bank accession numbers are shown in the Supplementary Table S1. Bootstrap values expressed as percentages of 1000 replications are given at the branches. The bar indicates an evolutionary distance of 0.1. The following color code highlights the different systematic groups.

Betaine biosynthesis is found in two major phylogenetic branches of *Cyanobacteria*, but ectoine biosynthesis is absent (Table 1, Figure 1). One branch is formed by *Aphanothece halophytica* (*Halothece* PCC7418), together with other *Chroococcales*. *Aphanothece halophytica* is a characteristic inhabitant of hypersaline environments and among the most halotolerant of *Cyanobacteria*. A gene cluster of the two methyltransferases (GMT and DMT) is present in these bacteria (Table 1). The BetT present in *Aphanothece halophytica,* which lacks choline-dependent betaine synthesis, is characterized as a specific transporter of betaine [18]. A second major branch of *Cyanobacteria* includes representatives of the heterogeneous groups of the *Synechococccus* and *Prochlorococcus* species and is phylogenetically quite distinct from the first branch (Figure 1). These Cyanobacteria have *proXWV* genes included in a cluster with GMT and DMT genes (Table 1).

The two groups are also distinguished by significant difference in the G + C content of the DNA. Representative strains of the *Prochlorococcus* group have a G + C content near 50 mol% for the *Prochlorococcus marinus* (strain MIT9313: 50.7 and strain MIT9303: 50.0%) and near 59% for *Synechococcus* (strain WH8102: 59.4% and strain WH8103: 59.5%). Much lower values are present in the *Halothece/Aphanothece* group: 42.9 mol% in *Aphanothece halophytica*, 41.1 mol% in *Euhalothece natronophila*, and 42.4 mol% in *Dactylococcopsis salina*. Although systematically assigned to the *Synechococcales*, *Dactylococcopsis salina* fits very well into the *Halothece* group, according to the gene repertoire and the phylogeny of the GMT sequence (Figure 1, Table 1) as well as the G + C content, which puts a question mark to its current taxonomic affiliation.

#### 3.1.2. Chlorobiaceae

As most of the green sulfur bacteria thrive in freshwater habitats, it is not surprising that they lack possibilities of synthesis of betaine and ectoine. The marine *Chloroherpeton thalassium* is the only one that can produce betaine from glycine (Table 1 and Figure 1). However, members of the genus *Prosthecochloris* and in particular *Prosthecochloris aestuarii* are also regularly found in brackish and marine coastal or saline habitats. They might cope with marine concentrations of salt by accumulation of trehalose, as shown to occur in *Prosthecochloris vibrioformis* DSM 260 (Pfennig 6030) and *Chlorobaculum thiosulfatophilum* DSM 249 (Pfennig 6230) [13]. If grown in marine media supplied with betaine, they can also accumulate the betaine [13]. Therefore, they have limited possibilities to cope with salt stress by accumulation of trehalose and uptake of betaine from the environment, to thrive at elevated salt concentrations. It is expected that they take advantage of betaine uptake when occurring in hypersaline habitats. A gene cluster annotated as *proVopuAB,AC* in *Prosthecochloris vibrioformis* DSM 260 (Table 1) presumably is a betaine transport system. The sequence of OpuAB is clearly distinct from those found in the *Chromatiaceae* and *Ectothiorhodospira* species, as well as from the ProW sequences found in other *Chromatiaceae* and *Halorhodospira* species and its ProV sequence is an outsider of the OpuAA branch (data not shown). This indicates that components of the transport system of *Prosthecochloris vibrioformis* might be related to an ancient ancestor of both the OpuA and the ProU transport systems, both of which might have evolved from a similar ancient ancestor. It would be interesting to study their catalytic properties and the evolutionary path of both systems.

#### 3.1.3. Chloroflexi

Genes for the synthesis of ectoine and betaine were not found in the *Chloroflexus* and *Roseiflexus* species (Table 1). Additionally, transport systems for osmotica are absent from the *Chloroflexus* species, which characterizes them as strict freshwater bacteria. An ABC transport system (annotated as *opuCBproXV* and an ABC transport protein) found in the *Roseiflexus castenholzii* and *Roseiflexus* species RS-1 (Table 1) might as well represent an ancient evolutionary form of osmolyte transport and betaine uptake from the environment. Thereby, the *Roseiflexus* species might gain limited possibility for osmotic adaptation. As these bacteria are among the oldest mat-forming phototrophic bacteria and *Roseiflexus* might be able to take up betaine or other osmolytes, they or relatives thereof are expected to be found in marine microbial mats. It would be interesting to see, whether marine relatives have possibilities of compatible solute biosynthesis. The species known and characterized so far are expected to have lost such possibilities.

#### 3.1.4. Marine Rhizobiales

Freshwater *Rhizobiales* (species of *Rhodoblastus*, *Rhodopseudomonas*, *Blastochloris*, *Rhodomicrobium*, and *Rhodoplanes*) lack both, biosynthesis genes for ectoine and betaine (from choline and glycine) as well as transport systems for these osmolytes (Table 2). In a group of marine *Rhizobiales* (*Fulvimarina pelagi, Hoeflea phototrophica, Rhodobium orientis, Afifella* species), the choline-dependent biosynthesis of betaine (BetABI) and a BetT transporter are present (Table 2). In addition, one or more copies/versions of the ProU system are found. Therefore, osmotic adaptation of these bacteria to the marine environment can be achieved by uptake of betaine or choline. In the absence of these compounds in the environment, other compatible solutes might be accumulated. For *Afifella marina* (formerly *Rhodopseudomonas marina*), the accumulation of trehalose was demonstrated [12].

#### 3.1.5. Marine and Halotolerant Rhodobacteraceae

The *Rhodobacteraceae* include freshwater, marine and halophilic species. With the exception of the freshwater *Rhodobacter* species, all of them have a complete gene cluster for ectoine biosynthesis (*ectRectABCask-ect*). In addition, the extremely halotolerant *Roseivivax halodurans*, *Roseivivax roseus*, *Rhodosalinus sediminis*, and *Roseovarius nitratireducens* (not *Roseivivax halotolerans* and *Roseovarius halotolerans*) have genes of betaine biosynthesis from glycine, with gene clusters of varying composition (Table 2). In these bacteria, the GMT and DMT methyltransferase genes are fused, as in some of the halophilic *Rhodospirillaceae* (see below). Most of the *Rhodobacteraceae* have several transport systems for osmolytes. All have the BetT transport system, some strains have multiple copies. With the exception of *Rhodobaculum claviforme* and *Rhodovulum imhoffii*, they have several ProU systems, which according to the ProW sequences are phylogenetically distinct (Table 2 and see below under Section 3.5).

#### 3.1.6. Acetobacteraceae

Phototrophic Acetobacteraceae are freshwater bacteria. *Rhodopila globiformis* lacks possibilities of synthesis and transport of betaine and ectoine, and *Paracraurococcus ruber* depends on external supply of such compounds and on transport via a ProU system (type W2) for osmotic adaptation (Table 2). *Acidiphilium* species, however, can produce ectoine and hydroxyectoine and in addition have a ProU (type W3) uptake system associated with the genes of betaine biosynthesis from choline (*proXWVbetBA*). *Acidiphilium* species are adapted to life in acidic freshwater habitats and their acidophilic nature should preclude their development in neutral/basic marine habitats. Therefore, the accumulation of ectoine and possibly also betaine might play a role in adaptation to highly acidic conditions rather than to saline habitats.

#### 3.1.7. Marine and Halophilic Rhodospirillaceae

Freshwater species of *Rhodospirillaceae* including species of *Pararhodospirillum*, *Rhodospirillum*, and *Phaeospirillum* lack ectoine and betaine biosynthesis (from glycine) and only some strains of *Rhodospirillum rubrum* can synthesize betaine from choline or have a ProU transport system (Table 2). On the other hand, marine and halophilic *Rhodospirillaceae* (*Rhodospira, Rhodovibrio, Roseospira, Roseospirillum*, and *Caenispirillum* species) are genomically well equipped with possibilities of osmotic adaptation. These species are adapted to moderately and extremely high salt concentrations. In particular, the *Rhodovibrio* species tolerate more than 3 M (up to 20%) NaCl. All of them synthesize ectoine (*Caenispirillum salinarum* also hydroxyectoine) and betaine. With the exception of *Rhodospira trueperi* and *Roseospirillum parvum*, they can also transform choline to betaine (*betABI*). Different to other *Rhodospirillaceae*, in the *Rhodovibrio* species, the GMT and DMT genes are not fused and form a gene cluster with MAT and SHAase. Their GMT gene is the only example of a B-type GMT gene in *Alphaproteobacteria*, and the *ectA* gene is not included in an *ectABC* cluster, as in almost all other phototrophic bacteria producing ectoine. In addition to *betT* (multiple), marine *Rhodospirillaceae* have one or more ProU transport systems. These are related to the *proVWX* type W1 (*proU* of *E. coli*), *proXWV* (type W2), or *proXVW* (type W4) systems. In *Caenispirillum salinarum*, a type W3 ProW system is present (Table 2).

#### 3.1.8. Halophilic *Rhodothalassium salexigens*

*Rhodothalassium salexigens* is a moderate halophilic and especially salt tolerant bacterium that grows at salt concentrations exceeding 3 M (20% NaCl). The outstanding properties of *Rhodothalassium salexigens* as distinct from all other phototrophic *Alphaproteobacteria* are demonstrated by sequences of the 16S rRNA gene and of the photosynthesis reaction center and bacteriochlorophyll biosynthesis genes [46]. These are in line with its recognition as a separate genus, family and order of the *Alphaproteobacteria* [47,48]. Ectoine biosynthesis is absent (Table 2). Betaine biosynthesis from glycine is possible and GMT sequences form a distinct lineage among those of the *Alphaproteobacteria* (Figure 1). In addition, BetT and a type W1 ProU transport systems are present.

#### 3.1.9. Chromatiaceae

Freshwater Chromatiaceae including *Chromatium*, *Allochromatium,* and *Thiocystis* species lack betaine and ectoine biosynthesis and corresponding transport systems (Table 3). Marine and halophilic Chromatiaceae can synthesize betaine from glycine and have the BetT transport system, but lack ectoine biosynthesis. With the exception of *Marichromatium gracile* and *Thiorhodococcus drewsii*, betaine biosynthesis from choline is absent. The group of marine representatives including the *Marichromatium, Thiorhodococcus,* and *Imhoffiella* species, *Thiocapsa marina* and *Thiocystis violacea* DSM 208 has an *opuA* gene cluster, but lacks the ProW permease protein of ProU. An exception is *Thiorhodococcus minor,* which has a ProU (type W1) instead of the OpuA system (Table 3) like all other marine and halophilic Chromatiaceae.

#### 3.1.10. Ectothiorhodospiraceae

According to phylogeny of 16S rRNA and photosynthetic reaction center genes, the *Ectothiorhodospira* and *Halorhodospira* species form two clearly separated branches that might even require a separation at the family level [46]. This clear separation is also reflected in different options for osmotic adaptation and in different lineages of normal as well as B-type GMT sequences (Table 3, Figure 1, see Section 3.2.2).

The *Ectothiorhodospira* species lack ectoine biosynthesis, but can synthesize betaine from glycine (GMT-DMT-MAT), with the exception of *Ectothiorhodospira mobilis* and *Ectothiorhodospira marismortui* also from choline (*betABIproX*). They have a gene cluster including genes of the OpuA and BetT transport systems (*betT-opuAA,AB,AC*) and additional *betT* gene copies (except *Ectothiorhodospira vacuolata* and *Ectothiorhodospira shaposhnikovii*), but lack the otherwise common ProU transporter (Table 3). An exception is the *Ectothiorhodospira magna*, which obviously lacks the biosynthetic capabilities and entirely depends on the uptake of betaine and ectoine (*betT-opuAA,AB,AC*) (Table 3). *Ectothiorhodosinus mongolicus* and *Thiorhodospira sibirica* are exceptions among the *Ectothiorhodospiraceae*. While the first lacks genes for biosynthesis of betaine and ectoine and only has ProU (type W1) and BetT transport systems, the latter has no annotated osmotic stress genes at all. This limits the possible adaptation to elevated salt concentrations and suggests that alternative mechanisms/solutes are used to cope with the salt in the environment. Sucrose, N-carbamoyl-L-glutamine amide, or N-acetyl-glutaminylglutamine amide are possible candidates that were found to accumulate in other purple sulfur bacteria, though in marine and halophilic species, they are found only in addition to betaine [12].

The *Halorhodospira* species are the most halophilic and halotolerant phototrophic bacteria and can thrive even in saturated salt solutions [1,2]. All *Halorhodospira* species have complete gene clusters for ectoine (*ectABC*) and betaine biosynthesis from glycine, but lack genes for transformation of choline to glycine (Table 3). They are the only *Gammaproteobacteria* to include adenosylmethionine synthetase (MAT), adenosylhomocysteinase (SAHase) and 5,10-methylene tetrahydrofolate reductase (MTHFR) into a gene cluster, together with the two methyltransferase genes (GMT-DMT-MAT-SAHase-MTHFR). They are well equipped with the transport systems for osmolytes and have multiple copies of BetT, the secondary transporter OpuD (except *Halorhodospira neutriphila*) and the ProU (type W1) transport system (except *Halorhodospira halochloris*). These transport systems assure that osmolytes leaking out of the cells at very high, several molar cytoplasmic concentrations can be regained by the cells, and are not wasted to the environment. Therefore, it is assumed that the available transport systems are able to take up betaine and ectoine.

### 3.2. Phylogeny of Glycine-Methyltransferase GMT

The methyltransferases that transform glycine to betaine, glycine and sarcosine methyltransferase (GMT), and dimethylglycine methyltransferase (DMT) are present in a wide range of phototrophic bacteria, *Alpha*-, and *Gammaproteobacteria*, *Cyanobacteria* and green sulfur bacteria, as revealed by genome analysis using the SEED facility of the RAST platform [39]; shown in Table 1, Table 2 and Table 3. A phylogenetic tree of GMT methyltransferases, which in addition to phototrophic bacteria includes data from the genomes of chemotrophic bacteria as well as from BLAST searches, is shown in Figure 1. Though many of the deep branching points are poorly resolved and not supported by bootstrap values, it is obvious that the phylogeny of GMT is well depicted in a number of major phylogenetic branches. The phylogenetic grouping correlates well with differences in the gene clusters involved in betaine biosynthesis (Figure 1). The following major groups and distinct phylogenetic lineages are recognized.

#### 3.2.1. Chromatiaceae

Among the phototrophic *Gammaproteobacteria*, marine and halophilic phototrophic *Chromatiaceae* and *Ectothiorhodospiraceae* species have the ability to produce betaine from glycine. Marine and halophilic species encoding this pathway of betaine biosynthesis are found in different phylogenetic lineages with (a) the halophilic species of the genus *Halochromatium*, *Lamprobacter modestohalophilus*, *Thiohalocapsa halophila* forming one branch and (b) the marine species of *Marichromatium, Thiocapsa*, *Thiorhodococcus*, *Thiorhodovibrio,* and *Thiocystis violacea* DSM 208, *Rhabdochromatium marinum*, together with *Thiococcus pfennigii* and *Thioflavicoccus mobilis*, forming a second one (Figure 1). In both of these branches of *Chromatiaceae,* including the separate lineages of *Thiohalobacter thiocyanaticus* and *Imhoffiella purpurea*, just the two methyltransferases (GMT and DMT) form a distinct gene cluster (Table 3).

#### 3.2.2. Ectothiorhodospiraceae

According to GMT sequences, species of *Halorhodospira* and *Ectothiorhodospira* form two clearly separated groups (Figure 1). In *Ectothiorhodospira*, species including *Thioalkalivibrio nitratireducens* and related species, GMT and DMT genes form a cluster together with the gene encoding S-adenosylmethionine synthetase (methionine adenosyl transferase, MAT), which is essential for performance of the methylation by providing the methyl donor S-adenosylmethionine. In the *Halorhodospira* species, in addition, the genes encoding S-adenosyl homocysteinase (SAHase) and 5,10-methylene tetrahydrofolate reductase (MTHFR) are included in this gene cluster (Table 3). The methionine synthase (MS) that completes the methionine cycle is located at a different location within the genome. The coordinated action of these enzymes is expected to allow optimal performance of betaine biosynthesis by providing the essential methyl groups and removing the byproduct S-adenosylhomocysteine, which strongly inhibits the reaction [15]. The chemotrophic *Arhodomonas aquaeolei* and *Nitrococcus mobilis* (only GMT-DMT cluster) form a distinct subbranch within the *Ectothiorhodospiraceae*.

There is a curiosity with the presence of a second additional single GMT gene within a few species of *Chromatiales*, which is phylogenetically distinct from the genes commonly found in other phototrophic bacteria, except the two *Rhodovibrio* species (Figure 1). We refer to these genes as the B-type methyltransferases in betaine biosynthesis, compared to the “common” system. GMT sequences of this B-type group form three lineages of phototrophic Gammaproteobacteria, (i) marine and halophilic *Chromatiaceae* species (*Halochromatium salexigens, Halochromatium glycolicum*, *Rhabdochromatium marinum* and *Thioflavicoccus mobilis*), (ii) *Halorhodospira* species (*Halorhodospira halophila*, *Halorhodospira neutriphila*), and (iii) *Ectothiorhodospira* species (*Ectothiorhodospira mobilis*, *Ectothiorhodospira marismortui*, *Ectothiorhodospira marina*) (Figure 1). In contrast, the B-type GMT genes of *Rhodovibrio sodomensis* and *Rhodovibrio salinarum* are the only ones for biosynthesis of betaine in these bacteria that are unique among *Alphaproteobacteria*. While this gene is included in a functional gene cluster and is quite likely active in the betaine synthesis of the *Rhodovibrio* species (GMT-DMT-MAT-SAHase), its role in the *Chromatiales* is unclear and it might represent an evolutionary relict or a backup.

#### 3.2.3. Cyanobacteria

Clearly two distinct branches of common GMT sequences of betaine biosynthesis are found in *Cyanobacteria* (Figure 1). The most divergent branch in the tree is represented by the *Prochlorococcus* group, including *Synechoccoccus* WH8102 and *Prochlorococcus marinus*. The second major phylogenetic branch of *Cyanobacteria*, the *Halothece/Aphanothece* group, is represented by the most prominent member of halophilic *Cyanobacteria*, *Aphanothece halophytica* (*Halothece* PCC7418), and several *Chroococcales*, including *Euhalothece natronophila* and *Rubidibacter lacunae*, but also *Dactylococcopsis salina,* which might be misclassified as a member of the *Synechococcales*.

#### 3.2.4. Alphaproteobacteria

The GMT sequences of *Alphaproteobacteria* represent a diverse major branch, which includes the species of *Sphingomonadales, Rhodothalassiales, Rhodobacterales,* and *Rhodospirillales*, but no *Rhizobiales* (Figure 1). The following distinct subbranches of *Alphaproteobacteria* are formed:

- *Rhodospirillaceae*, including *Caenispirillum salinarum*, *Rhodospira trueperi*, *Roseospira navarrensis*, *Roseospira marina*, and *Roseospirillum parvum* (but not the *Rhodovibrio* species) have a fused GMT/DMT gene of the methyltransferases included in a small cluster with S-adenosylmethionine synthase (MAT).
- *Rhodothalassium salexigens*, which represents the most distant line to all other *Alphaproteobacteria*, has a small gene cluster with just the two methyltransferases.
- *Sphingomonadales* include *Novosphingobium malayensis*, *Erythrobacter litoralis*, *Altererythrobacter atlanticus*; in *Erythrobacter litoralis* just the two methyltransferases form a small gene cluster.
- *Hyphomonadaceae* include the *Glycocaulis and Marinicauda* species with a small gene cluster of the two methyltransferases only.
- *Rhodobacteraceae* included in the study, as indicated in Figure 1, form a subbranch together with *Thalassobaculum litoreum* and *Thalassobaculum salexigens* (according to 16S rRNA phylogeny forming a branch with the *Oceanobaculum* species at an almost equal distance to the *Rhodobacteraceae* and *Rhodospirillaceae* species; data not shown). A fused GMT/DMT gene is associated with the MAT gene in *Roseivivax halodurans*, with the MS-MAT genes in *Rhodosalinus sediminis*, and with the MTHFR-MS-MAT-SAHase genes in *Roseivivax roseus* and *Roseivarius nitratireducens* (Table 2).

#### 3.2.5. Actinobacteria

The Actinobacteria are the only major group of chemotrophic bacteria that show betaine biosynthesis from glycine. They form a distinct branch distantly related to *Chromatiales* and *Chloroherpeton* (Figure 1). In *Actinopolyspora halophila*, for example, a fused GMT/DMT gene is included in a gene cluster with MAT, MTHFR, and SAHase (Table 4).

#### 3.2.6. Chlorobiaceae

Among the green sulfur bacteria, betaine biosynthesis is found only in *Chloroherpeton thalassium*. The GMT sequence forms a separate line that associates distantly with those of the halophilic *Chromatiales* and *Actinobacteria* (Figure 1). Both methyltransferase genes are found in a small cluster together with the BetT transport system (Table 1).

#### 3.2.7. Pirellulaceae

The two methyltransferases of betaine biosynthesis (GMT, DMT) are also found in the chemotrophic *Rhodopirellula europaea* and *Rubripirellula obstinata* (*Pirellulaceae*). Phylogenetically, these GMT sequences form a distinct branch distantly associated with *Ectothiorhodospiraceae*, though with low confidence. In *Rhodopirellula europaea*, the *betT* gene forms a cluster with the two methyltransferases (*GMT-DMT-betT*).

### 3.3. Phylogeny of EctB

**Figure 2:**
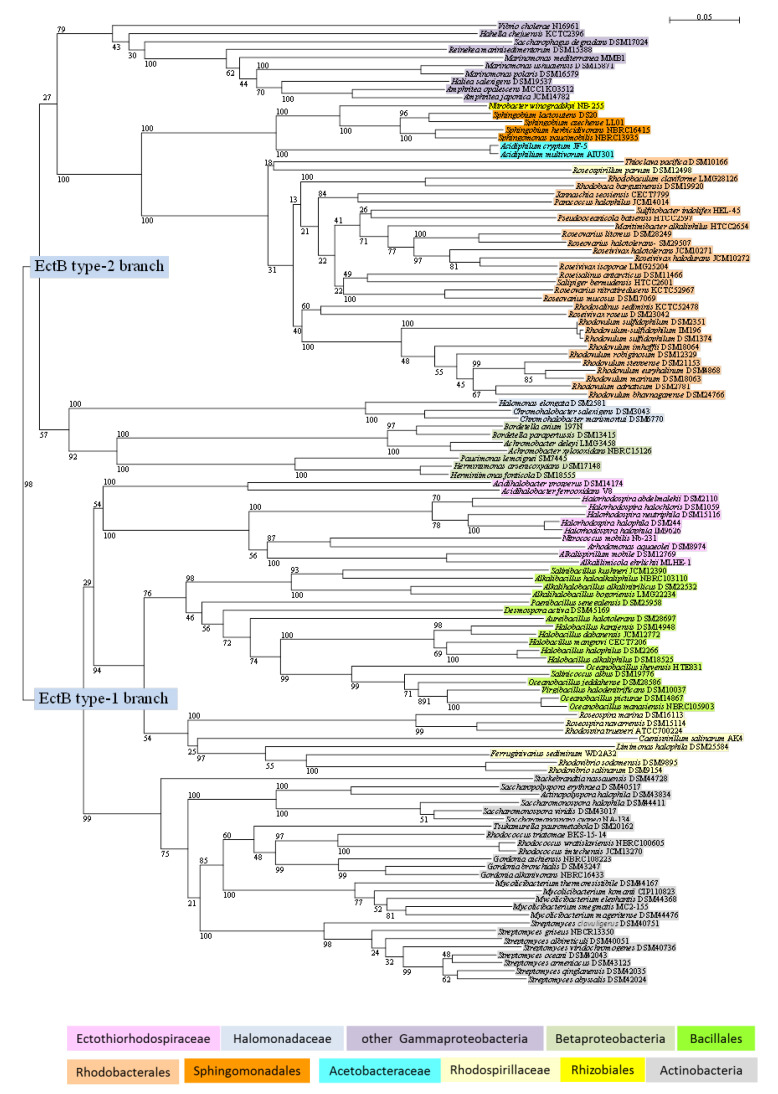
The phylogeny of ectoine biosynthesis on the basis of sequences of diaminobutyrate-pyruvate aminotransferase EctB is shown in a neighbor-joining tree. Sequences and gene bank accession numbers are shown in the Supplementary Table S2. Bootstrap values expressed as percentages of 1000 replications are given at the branches. The bar indicates an evolutionary distance of 0.05. The following color code highlights different systematic groups.

The ability for ectoine biosynthesis is found in several phylogenetic distant lineages of anoxygenic phototrophic bacteria. Two major phylogenetic branches (type-1 and type-2 EctB sequences) can be distinguished, which also show differences in the *ect* gene cluster structure (Figure 2).

*Gammaproteobacteria* are found in both branches—representatives of *Ectothiorhodospiraceae* in the EctB type-1 branch and representatives of *Oceanospirillales*, *Cellvibrionales*, and *Vibrionales* in the EctB type-2 branch. According to genome analysis of the selected species and also BLAST search with representative EctB sequences, ectoine biosynthesis is absent from all tested *Cyanobacteria*, from *Heliobacteria*, *Chlorobi*, *Chloroflexi*, *Chloracidobacterium*, as well as from all studied phototrophic *Betaproteobacteria*, *Rhizobiales*, *Chromatiaceae*, and the *Ectothiorhodospira* species (Table 1, Table 2 and Table 3).

As shown in Figure 2, a first major phylogenetic branch (EctB type-1) contains four clearly separated groups: (1) the *Ectothiorhodospiraceae*, (2) *Rhodospirillaceae* including the non-phototrophic relatives, (3) *Bacillales*, and (4) *Actinobacteria*. This branch is characterized by a gene cluster lacking the regulatory *ectR* gene and the *ask_ect* gene.

- The *Ectothiorhodospiraceae* form a branch with representatives of the genera *Halorhodospira*, *Alkalilimnicola*, *Alkalispirillum*, *Nitrococcus*, *Arhodomonas*, and *Acidihalobacter*, but lack species of *Ectothiorhodospira*, *Thiorhodospira*, and *Ectothiorhodosinus*, in which ectoine biosynthesis is absent. A cluster of the *ectABC* genes that lacks the regulatory gene is found in the extremely halophilic *Halorhodospira* species.
- The marine and halophilic *Rhodospirillaceae* also have an *ectABC* gene cluster and form deeply branching separate lineages with *Rhodospira trueperi* and the *Roseospira* species in one, *Rhodovibrio sodomensis* and *Rhodovibrio salinarum* and the non-phototrophic *Ferruginivarius sediminum* and *Limimonas halophila* in another, and *Caenispirillum salinarum* in a third lineage (Figure 2).
- Different lineages of the *Bacillales* branch are represented by species of the genera *Halobacillus*, of *Oceanobacillus* (including *Salinicoccus albus* (*Staphylococcaceae*) and *Virgibacillus halodenitrificans*), of *Salinibacillus, Alkalibacillus* and *Alkalihalobacillus*. Distinct separate lines of *Aureibacillus halotolerans* (*Bacillaceae*), *Paenibacillus senegaliensis* (*Paenibacillaceae*) and *Desmospora activa* (*Thermoactinomycetaceae*) are found (Figure 2).
- The branch of *Actinobacteria* shows distinct subbranches of *Streptomyces*, *Mycolicibacterium*, *Gordonia*, and *Rhodococcus* (associated with the *Tsukamurella paurometabola*) species, and of *Saccharomonospora*, *Actinopolyspora*, and *Saccharopolyspora* species (Figure 2). Distinct from these and as an outsider of the group is *Stackebrandtia nassauensis*. Common among many of the *Actinobacteria* is the ability to form hydroxyectoine, as demonstrated here by genome analysis of *Mycolicibacterium thermoresistibile*, and BLAST search with the EctD sequences from these bacteria (data not shown).

A second major phylogenetic branch (EctB type-2) is characterized by the presence of an extended gene cluster for ectoine biosynthesis, often including the regulatory gene (*ectR*) and a specific isoenzyme of aspartokinase (*ask_ect*). The EctB type-2 group shows considerable deeper branching points and is phylogenetically even more diverse compared to the EctB type-1 group (Figure 2).

The *Alphaproteobacteria* form two related subbranches. One of these is represented by *Rhodobacterales*, with numerous anaerobic and aerobic phototrophic bacteria, as well as chemotrophic relatives that are unable to perform photosynthesis and has an extended *ask_ect_ectABCR* gene cluster. Species of *Rhodovulum* and the related genera *Roseivivax*, *Roseovarius*, *Rhodosalinus*, and *Roseisalinus* are included (Table 2, Figure 2). However, in the related *Rhodobacter* species, ectoine biosynthesis and the *ectABCR* genes are absent. One distinct separate lineage is formed by *Rhodobaca barguzinensis* and *Rhodobaculum claviforme*. The marine *Roseospirillum parvum* is the only representative of *Rhodospirillaceae* within the type-2 group. Its gene cluster includes the regulatory gene (*ectABCR*) but lacks the *ask_ect* gene. However, this species and *Thioclava pacifica* (*Rhodobacteraceae*) are outsider of this subbranch.

A second subbranch of *Alphaproteobacteria* is distantly associated with the *Rhodobacterales* branch. It includes the aerobic phototrophic *Acidiphilium multivorum* (*Acetobacteraceae*, *Rhodospirillales*) and *Acidiphilium cryptum* (non-phototrophic), which also encode the formation of hydroxyectoine and have *ectD* included in the gene cluster *ectRABCDask_ect* (Table 2). The related phototrophic *Acetobacteraceae Rhodopila globiformis* and *Paracraurococcus ruber* are unable to produce ectoine (Table 2). A second lineage of this subbranch contains *Nitrobacter winogradskyi* (*Rhizobiales*) and representatives of the *Sphingomonadales*.

Although none of the phototrophic *Betaproteobacteria* included in this study was able to synthesize ectoine, in several chemotrophic *Betaproteobacteria* (*Burkholderiales*) EctB is present and a distinct branch of type-2 EctB sequences is formed. Two separate lineages include representatives of *Alcaligenaceae* (*Achromobacter* and *Bordetella* species) in one, and the *Paucimonas* (*Burkholderiaceae*) and *Herminiimonas* (*Oxalobacteraceae*) species in another lineage (Figure 2). An *ectRABCD* gene cluster was found in the genomes of *Achromobacter xylosoxidans*, *Bordetella avium*, *Herminiimonas arsenicoxydans*, and *Paucimonas limoigeni* (Table 4).

Two branches of *Gammaproteobacteria* are found among the EctB type-2 sequences. One is represented by *Halomonadaceae* with *Halomonas elongata* and *Chromohalobacter salexigens* and related species (*ectABC*/*ectD*), and associates distantly with the sequences of *Betaproteobacteria*. The second one is poorly associated and is quite distant to the branch of *Alphaproteobacteria*. It includes representatives of *Oceanospirillaceae* (*Marinomonas* and *Amphritea* species) and deeply branching lines with representatives of other families (Table 4, Figure 2). There is a considerable variation of the composition of the *ect* gene cluster in this branch (*Marinomonas mediterranea*: *ectAB*C/*ectR*/*ask_ect*; *Hahella chejuvensis*: *ectABCD*; *Haliea salexigens*: *ectRABCask_ect*), as shown in Table 4.

### 3.4. Betaine Synthesis from Choline—Distribution of Bet Genes in Phototrophic Bacteria

An alternative and independent pathway of betaine synthesis that starts from choline exists in a number of bacteria [49]. It depends on an external source of choline that needs to be taken up by the cells and is then converted into betaine. Thus, the availability of choline is a crucial factor that eliminates this pathway from consideration, as an important and independent option to adapt to high salt concentrations. Though this pathway offers a good chance for bacteria living in eutrophic locations rich in biomass and provide choline as the source, such habitats with extremes of salt concentrations are almost devoid of higher developed eukaryotes that could produce and release choline. Here, mass developments of halophilic microorganisms that would be the primary colonizers could be a possible source under such conditions. If the presence of this pathway is compared with the ability to cope with even low (marine) salt concentrations, it is then obvious that it does not play a primary role in conquering marine and hypersaline habitats. Most marine species that can perform the choline pathway, are not dependent on this pathway, but have alternative options of compatible solute biosynthesis. Examples are *Rhodobaca barguzinensis* (produces also ectoine), *Rhodovibrio* species (also have the ectoine and glycine-dependent pathway), *Marichromatium gracile* (also has the glycine-dependent pathway), and *Ectothiorhodospira* species (have the glycine-dependent pathway) (see Table 2 and Table 3). The only marine species that exclusively rely on the choline-dependent pathway of biosynthesis for betaine synthesis (in addition to several transport systems) are members of the *Rhizobiales* including *Fulvimarina pelagi*, *Hoeflea phototrophica*, *Rhodobium orientis*, and the *Afifella* species (Table 2). It is also found in some freshwater bacteria, such as *Rhodopseudomonas palustris*, *Rhodospirillum rubrum*, and *Rhodobacter* species.

Quite interestingly, the choline-dependent pathway of betaine synthesis is also an additional option in a few extremely halotolerant species, which are capable of betaine synthesis from glycine and of ectoine synthesis. These include a few *Rhodobacteraceae* (species of *Roseivivax*, *Rhodosalinus*, and *Roseovarius*), as well as the *Rhodovibrio* species (Table 2).

### 3.5. Transport System for Uptake of Glycine Betaine and Choline

All marine and halophilic phototrophic bacteria have one or more transport systems for betaine or choline. This underlines the importance of transport to gain or regain osmolytes for successful adaptation to elevated salt concentrations. In contrast, freshwater phototrophic bacteria not only lack possibilities of biosynthesis but also of common transport systems for betaine, ectoine, and related osmolytes. While there are no obvious correlations between these transport systems and the biosynthetic pathways present, various phylogenetic groups have characteristic sets of transport systems for betaine, choline, and possibly ectoine and related osmolytes.

**Figure 3:**
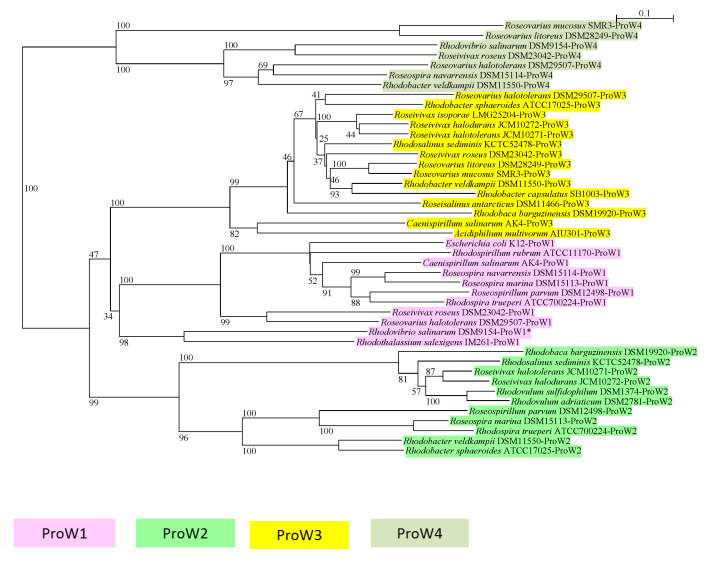
The phylogeny of the permease protein ProW of the L-proline/glycine betaine transport system ProU is shown in a neighbor-joining tree. The ProW1-ProW4 gene clusters are defined with Table 1. Sequences and gene bank accession numbers are shown in Supplementary Table S3. Bootstrap values expressed as percentages of 1000 replications are given at the branches. The bar indicates an evolutionary distance of 0.1. The following color code is used for the different ProW sequence types.

The most common and highly variable system is the ABC transporter ProU, which is present in different distinct variants and in different association with other genes. Often multiple forms are found in one and the same bacterium (Figure 3, Table 1, Table 2 and Table 3).

According to the ProW sequences, at least four distinct types of this transporter can be distinguished (Figure 3). One separate *proVWX* gene cluster is related to the *E. coli proU* cluster (type ProW1). Another separate *proXWV* cluster represents a second phylogenetic group (type ProW2). Phylogenetically distinct is a third group (type ProW3), which has *proXWV* genes associated with a cluster of genes for choline-dependent betaine biosynthesis (*betABI-proXWV*), e.g., in *Rhodosalinus sediminis*. A fourth group with a particularly large ProW sequences (type ProW4) is clearly distant to the first three groups. Finally, a gene cluster including a YehZ betaine binding protein is especially found in some species restricted to freshwater habitats (data not shown). In fact, YehZYXW mediated activity in *E. coli* was inhibited at increased salinity and it was concluded that this transport system is not relevant to osmotic protection [50]. A branch of sequences of ProW of this type of transport system is distantly related to the four others and includes sequences of the *E. coli* YehW (data not shown). This was not considered in the phylogenetic tree of ProW sequences (Figure 3). In this tree of selected ProW sequences, the presence of several sequence types is demonstrated in *Rhodobacter veldkampii* (types W2, W3, W4), *Rhodobaca barguzinensis* (types W2, W3), *Roseovarius halotolerans* (types W1, W3, W4), and others. Though sequence variations might suggest possible differences in catalytic properties, gene regulation or substrate specificities between the 4 types of ProU transport systems, these are yet to be demonstrated in future studies.

Common to most marine and halophilic phototrophic bacteria, no matter whether they synthesize betaine or ectoine, is the BetT secondary transporter, which is known as a specific choline transport system in *E. coli*, but is shown to specifically transport betaine in *Aphanothece halophytica* [18].

Another secondary transporter for glycine betaine, OpuD, is found in the freshwater Betaproteobacteria *Rhodoferax fermentans* and *Rubrivivax gelatinosus*, as only a transport system for betaine.Whereas, it is present in the marine *Caenispirillum salinarum* as well as in the extreme halophilic *Halorhodospira* species, both of which synthesize betaine and ectoine, (not in *Hlr. neutriphila*) together with BetT and ProU (type W1) (not in *Hlr. halochloris*). The only species of *Ectothiorhodospira* that encodes OpuD is *Ectothiorhodospira haloalkaliphila*.

The ABC transporter OpuA was found together with BetT in a small group of marine Chromatiaceae producing betaine (*Thiocapsa*, *Thiocystis*, *Marichromatium*, *Imhoffiella*, and *Thiorhodococcus* species), and also in all *Ectothiorhodospira* species. In all of these latter species, *betT* clusters with the *opuA* genes (*betT*-*opuAA,AB,AC*) and in a few Chromatiaceae, a copy of *betT* also specifically associates with the *opuAC* gene (Table 3). In a few cases, a chimera of the *proU* and *opuA* genes occur. In marine *Prosthecochloris vibrioformis*, this is *proVopuAB,AC*, in *Rhodovibrio salinarum* and also in two Chromatiaceae (*Rhabdochromatium marinum* and *Thiorhodovibrio winogradskyi*) this is *proVWopuAC*. Furthermore, a chimera between *opuC* and *proU* (*opuCB-proXV*) is annotated in the *Roseiflexus* species.

### 3.6. Evolutionary Considerations

From an evolutionary point of view, the primary adaptation to high solute concentrations clearly requires a biosynthesis of compatible solutes. Already in the early prokaryote era the ability for biosynthesis and accumulation of organic solutes was a prerequisite for archaic eubacteria to conquer saline and hypersaline environments. Inorganic ions and common simple organic metabolites might have allowed a basic osmotic adaptation in freshwater and marine bacteria. Potassium very likely is a suitable candidate and is responsible for a kind of basic osmotic adaptation in many bacteria in which it accumulates together with glutamic acid, and thereby contributes to the overall osmotic balance [7]. In the extremely halophilic Archaea, the *Halobacteria*, it is even the primary osmolyte and accumulates to several molar concentrations [51,52]. In freshwater bacteria such as *E. coli*, its uptake or release makes possible rapid responses to small changes in osmotic conditions. For osmotic adaptation of marine and halophilic eubacteria, however, it is not important.

In marine eubacteria, a number of non-charged neutral organic molecules contribute to different degrees in achieving osmotic balance. As was shown for *Cyanobacteria*, the degree of salt tolerance depends on the kind of osmolytes that are accumulated. Only limited osmotic adaptation to lower ranges of salt concentrations is possible by carbon compounds such as sugars (sucrose, trehalose), glucosylglycerol, and others [1,6,7,11,12,13]. The advantage of accumulating these compounds is the comparable cheap biosynthesis and the absence of nitrogen as one of the most severe limiting elements in the environment. The disadvantage is their comparatively low compatibility or solubility. For example, trehalose has a solubility of approximately 2 M, ectoine of 3.87 M, and glycine betaine of 13.6 M. The extreme compatibility of betaine, which goes hand in hand with its excellent solubility, and the almost perfect ability to protect macromolecules against denaturation even at very high concentrations, make betaine the top compatible solute. The excellent osmotic protection of betaine is also reflected in the finding, that the phototrophic bacteria that have most successfully adapted to extremely high salt concentrations accumulate betaine as compatible solute through de novo biosynthesis from glycine. In fact, a striking dependence on betaine synthesis for adaption, to live in extreme and even moderate salt concentrations as found in this study, points out that the kind of osmolytes accumulated in a critical way determine the success of salt adaptation. Therefore, the establishment of betaine biosynthesis is expected to represent an evolutionary breakthrough in osmotic adaptation of eubacteria to high salt concentrations, and is considered to be a prerequisite to conquer highly saline environments.

Nonetheless, the synthesis and accumulation of high concentrations of betaine is a costly process. Though glycine, the immediate biosynthetic precursor of betaine, is a common intermediate of central metabolic processes and can be transformed to betaine with a few enzymatic steps, the three methylation steps have a high energy demand. Additionally, the accumulation of several molar concentrations can be easily limited by the availability of nitrogen. Therefore, bacteria that have no shortage in energy and nitrogen supply, such as phototrophic bacteria, which use sunlight as an energy source and are capable of fixing dinitrogen, are likely to be the first and also to be the most successful in conquering highly saline habitats. In fact, mass developments of phototrophic bacteria regularly occur in salt and soda lakes, as well as in coastal lagoons, and cause colored blooms [1,7,53,54,55].

Compared to betaine, the accumulation of ectoine has two major and significant disadvantages. The physiological disadvantage of ectoine is its low solubility compared to betaine, which limits its accumulation and protective action at hypersaline salt concentrations. Its ecological disadvantage is the content of 2 nitrogen atoms in the molecule, which doubles the requirement for nitrogen compared to betaine. This restricts accumulation to environments with a high content of combined nitrogen compounds, unless nitrogen can be supplied by nitrogen fixation. In this case, the energy requirement for osmotic adaptation is further increased, because twice as much nitrogen has to be fixed, compared to betaine synthesis. Phylogenetically, it appears as a late event compared to betaine biosynthesis. The most deeply branching points within both type-1 and type-2 EctB sequences are those of the Gammaproteobacteria, forming one branch in the type-1 group and two branches in the type-2 group. Quite remarkably, also the structure of the *ect* gene cluster is much more variable in Gammaproteobacteria compared to other groups. In addition to the *ectABC* genes, either *ectD* or *ask_ect* or *ectR*, or combinations thereof might be present, but might not be part of the gene cluster (Table 3). Therefore, it is assumed that the two types of EctB sequences have their origin in ancestors of the *Gammaproteobacteria*, which might also represent the most ancient ectoine producers. Both phylogenetic lineages might have separated early and given rise to the two independent major branches. Today, the two most prominent and best studied representatives of the two branches that have evolved are found in the Gammaproteobacteria: *Halorhodospira halochloris* (type-1) and *Halomonas elongata* (type-2). Among phototrophic bacteria, ectoine biosynthesis is restricted to a few distinct groups, with representatives of *Gammaproteobacteria* (*Halorhodospira* species) and *Alphaproteobacteria* (*Rhodobacterales* and *Rhodospirillales*).

## 4. Conclusions

The comprehensive analysis of more than 130 genomes of phototrophic bacteria gives insight into their ability to synthesize the compatible solutes betaine and ectoine, and to take up these compounds. The potential to accumulate these compatible solutes and the kind of solute accumulated, clearly define the range of salt concentrations and the habitats where these bacteria can develop. The data suggest that betaine is the primary compatible solute at high salt concentrations and the most ancient one in evolutionary terms. All halophilic phototrophic bacteria rely on betaine synthesis, and only few of them have additional options of ectoine biosynthesis, or betaine synthesis from choline. Ectoine synthesis as a sole compatible solute is only found in some marine bacteria, in particular *Rhodobacteraceae* and the acidiphilic *Acidiphilum* species. This is in accord with its potential to achieve osmotic protection in moderately halotolerant marine bacteria. As information on the presence of choline in marine and hypersaline habitats is missing, it is unclear whether the transformation of choline to betaine is of relevance for osmotic adaptation in these environments. Only few marine species of the *Rhizobiales,* exclusively rely on this pathway, but in addition have several transport systems (betT, ProU) to take up betaine and ectoine from the environment. Therefore, these bacteria are more limited than others to adapt to marine salt concentrations, and their occurrence is restricted to habitats where appropriate solutes are available.

The possibility to take up and accumulate compatible solutes or their precursors from the environment and also from the culture media, was been largely neglected in previous studies on salt relations of marine and halophilic species. In order to consider osmotic adaptation in an ecological context and to characterize salt relations of individual species, the potential of uptake needs to be considered. In complex microbial communities such a microbial mats, the study of synthesis, release and uptake at the community level would be rewarding and would shed light on possible complex syntrophic interactions based on the exchange of compatible solutes between members of the community. In view of the variety and the presence of multiple transport systems in several species, characterization of their catalytic properties and regulation is necessary to identify their functional roles. Quite rewarding from both an evolutionary point of view and from a functional context, should be the analysis of chimeric transport systems as they occur in *Prosthecochloris vibrioformis* (*proVopuAB,AC*) and in the *Roseiflexus* species (*opuCBproXV*).

Despite the fact that synthesis and accumulation of betaine is common to all known halophilic phototrophic bacteria, there is a considerable variation in gene arrangement and formation of gene clusters. In addition, fused GMT-DMT genes occur in some groups of the studied bacteria and a second type called the B-type of GMT sequences is found in several species. The phylogeny of the biosynthesis pathway (of GMT), suggests that the roots are manifested early in bacterial evolution and are most likely before diversification of bacteria as we know today. The recognition of two major phylogenetic branches of *Cyanobacteria* and their relations to others suggests that they represent one of the most ancient betaine-producing bacterial phyla and betaine biosynthesis and might have originated in one of their early ancestor. In another very ancient phylum of phototrophic bacteria, the *Chlorobi* (green sulfur bacteria), betaine biosynthesis was found only in *Chloroherpeton thalassium*. This is one of the most ancient representatives of green sulfur bacteria that is known and the deeply rooted branch of its GMT sequence points out that it might represent one of the most ancient betaine producers as well. Other green sulfur bacteria such as the *Chlorobium* and *Chlorobaculum* species and their relatives might have lost the capability of betaine synthesis during adaptation to freshwater habitats. Most remarkable is the occurrence of B-type GMT sequences, which are phylogenetically distant to all other GMT sequences, and might represent a much older system of betaine biosynthesis. Such a GMT gene is included in a gene cluster for betaine biosynthesis only in the *Rhodovibrio* species. Therefore, studies of betaine biosynthesis of the *Rhodovibrio* species should be quite rewarding.

In addition to these few examples, data are presented form a comprehensive basis for more detailed studies on osmotic adaptation of phototrophic bacteria.

## Figures and Tables

**Table 1:** Genes and gene clusters of betaine and ectoine biosynthesis as well as relevant transport systems of phototrophic *Acidobacteria* (*Chloracidobacterium*), *Chlorobi*, *Chloroflexi*, *Cyanobacteria*, *Heliobacterium modesticaldum*, *Gemmatimonas phototrophica* and *Betaproteobacteria* are shown together with salt responses and their systematic affiliation ^a,b,c^.

Gene Repertoire of Various Orders of Phototrophic Bacteria for Osmotic Adaptation								
Family	Species	Strain	Salt	Betaine	biosynthesis	Osmolyte transport		
			response	from glycine	from choline			
				GMT-DMT	betAB	betT	opuA /opuC	proW1
							opuD	proW2
**Acidobacteria/Acidobacteriales**								
Acidobacteriaceae	*Chloracidobacterium thermophilum*	B-G2	F	o	o	o	o	o
								
**Chlorobi/Chlorobiales**								
**Chlorobiaceae**	*Chlorobaculum thiosulfatophilum*	DSM 249	F	o	o	o	o	o
Chlorobiaceae	*Chlorobium limicola*	DSM 245	F	o	o	o	o	o
Chlorobiaceae	*Chlorobium phaeovibrioides*	DSM 265	F	o	o	o	o	o
Chlorobiaceae	*Chloroherpeton thalassium*	ATCC 35110	M	GMT-DMT-betT	o	o	o	o
Chlorobiaceae	*Prosthecochloris aestuarii*	DSM 271	M	o	o	o	o	o
Chlorobiaceae	*Prosthecochloris vibrioformis*	DSM 260	M	o	o	o	proVopuAB,AC	o
								
**Chloroflexi/Chloroflexales**								
Chloroflexaceae	*Chloroflexus aggregans*	DSM 9485	F		o		o	o
Chloroflexaceae	*Chloroflexus aurantiacus*	J-10-fl	F	o	o		o	o
Roseiflexaceae	*Roseiflexus castenholzii*	DSM 13941	F	o	o		opuCB-proXV	o
Roseiflexaceae	*Roseiflexus* sp.	RS-1	F	o	o		opuCB-proXV	o
								
****Cyanobacteria/Synechococcales****								
Prochloraceae	*Prochlorococcus marinus*	MIT 9313	M	GMT-DMTproVWX	o	o	o	o
Synechococcaceae	*Synechococcus* species	WH8102	M	GMT-DMTproVWX	o	o	o	o
Synechococcaceae	*Dactylococcopsis salina*	PCC 8305	H	GMT-DMT	o	o	o	W1
								
**Cyanobacteria/Chroococcales**								
Aphanothecaceae	*Halothece* sp./*Aphanothece halophytica*	PCC 7418	H	GMT-DMT	o	/betT/	o	W1
Aphanothecaceae	*Euhalothece natronophila*	Z-M001	H	GMT-DMT	o	/betT/	o	W1
								
**Firmicutes/Clostridiales**								
Heliobacteriaceae	*Heliobacterium modesticaldum*	Ice1	F	o	o	o	o	o
								
**Gemmatimonadetes/Gemmatimonadales**								
Gemmatimonadaceae	*Gemmatimonas phototrophica*	AP64	F	o	o	o	o	o
								
**Betaproteobacteria/Burkholderiales**								
Burkholderiaceae	*Polynucleobacter duraquae*	MWH-MoK4	F	o	o	o	o	o
Comamonadaceae	*Rhodoferax antarcticus*	DSM 24876	F	o	o	o	o	W2
Comamonadaceae	*Rhodoferax fermentans*	DSM 10138	F	o	o	o	opuD	o
uncl. Burkholderiales	*Rubrivivax gelatinosus*	IL144	F	o	o	o	opuD	o
uncl. Burkholderiales	*Rubrivivax gelatinosus*	DSM 1709	F	o	o	o	opuD	o
uncl. Burkholderiales	*Rubrivivax gelatinosus*	IM 151	F	o	o	o	opuD	o
uncl. Burkholderiales	*Rubrivivax gelatinosus* 155	DSM 149	F	o	o	o	opuD	o
								
Betaproteobacteria/Rhodocyclales								
Rhodocyclaceae	*Rhodocyclus purpureus*	TEM	F	o	o	o	o	o
Rhodocyclaceae	*Rhodocyclus tenuis*	IM 230	F	o	o	o	o	o

^a^ Variants of the transport system are abbreviated W1 = proVWX, W2 = proXWV, W3 = proXWV-bet, W4 = proXVWlarge for proU and opuA = opuAA,AB,AC. The code for salt responses (growth optimum/tolerance) is F = freshwater species (<1% NaCl), M = marine species (1–7%/<8–9%), M/H = marine species with elevated salt tolerance (1–8%/>10–20%), and H = moderate to extreme halophilic species (>6–25%/>15–>25%). As far as possible, the gene clusters are given and “/” denotes a separate locus of the genes in the genome. The genome accession numbers of GenBank are shown in the Supplementary Table S4. ^b^ Ectoine biosynthesis absent, ^c^ proW3, and ProW4 are absent. Color shades indicate different types of gene and gene associations: betaine synthesis from glycine (blue) and from choline (green), transport with betT (light-lila), opuA,C and D (shades of beige-brown), proU-W1 and W2 (shades of green); also marine (blue) and halophilic (rose-pink) growth response of the bacteria.

**Table 2:** Genes and gene clusters of betaine and ectoine biosynthesis as well as relevant transport systems of phototrophic *Alphaproteobacteria* ^a^.

Gene Repertoire of Phototrophic Alphaproteobacteria for Osmotic Adaptation												
Family	Species	Strain	Salt	Ectoine	Betaine	biosynthesis	Osmolyte transport					
			response	biosynthesis	from glycine	from choline						
				ectABC	GMT-DMT	betAB	betT	opuA	proW1	proW2	proW3	proW4
**Rhizobiales**								opuD				
Beijerinckiaceae	*Methylocella silvestris*	BL2	F	o	o	o	o	o	o	o	o	o
Beijerinckiaceae	*Rhodoblastus acidophilus*	DSM 137	F	o	o	o	o	o	o	o	o	o
Beijerinckiaceae	*Rhodoblastus sphagnicola*	DSM 16996	F	o	o	o	o	o	o	o	o	o
Bradyrhizobiaceae	*Bradyrhizobium oligotrophicum*	S58	F	o	o	o	o	o	o	o	o	o
Bradyrhizobiaceae	*Rhodopseudomonas palustris*	DSM 126	F	o	o	/betA/ 3x /betB/	o	o	o	o	o	o
Bradyrhizobiaceae	*Rhodopseudomonas pseudopalustris*	DSM 123T	F	o	o	o	o	o	o	o	o	o
Hyphomicrobiaceae	*Blastochloris tepida*	GI	F	o	o	o	o	o	o	o	o	o
Hyphomicrobiaceae	*Blastochloris viridis*	DSM 133	F	o	o	o	o	o	o	o	o	o
Hyphomicrobiaceae	*Rhodomicrobium vannielii*	ATCC 17100	F	o	o	o	o	o	o	o	o	o
Hyphomicrobiaceae	*Rhodoplanes elegans*	DSM 11907	F	o	o	o	o	o	o	o	o	o
Aurantimonadaceae	*Fulvimarina pelagi*	HTCC2506	M	ectB/	o	betABCIproXWV	/betT/ 3x	o	W1	W2	W3	o
Phyllobacteriaceae	*Hoeflea phototrophica*	DFL-43	M	o	o	betABCI	/betT/	o	o	W2	o	W4
Rhodobiaceae	*Afifella marina* 125/2	IM 162	M	o	o	proXbetIA /betB/	/betT/	o	W1	o	o	o
Rhodobiaceae	*Afifella marina* 125/4)	IM 163	M	o	o	proXbetIA /betB/	/betT/	o	W1	o	o	o
Rhodobiaceae	*Afifella marina* 985, 126 (166)	DSM 2698	M	o	o	proXbetIA /betB/proWV	/betT/	o	W1	o	W3	o
Rhodobiaceae	*Afifella pfennigii*	DSM 17143	M	o	o	proXbetIA /betB/	/betT/	o	W1	o	o	o
Rhodobiaceae	*Rhodobium orientis*	DSM 11290	M	o	o	betABIbetCproXWV	/betT/	o	o	W2	W3	o
**Rhodobacterales**												
Rhodobacteraceae	*Rhodobacter capsulatus*	SB 1003	F	o	o	betABIproXWV		o	o	o	W3	o
Rhodobacteraceae	*Rhodobacter sphaeroides*	ATCC 17025	F	o	o	betABIproXWV		o	o	W2	W3	o
Rhodobacteraceae	*Rhodobacter veldkampii*	DSM 11550	F	o	o	betABIproXWV		o	o	W2	W3	W4
Rhodobacteraceae	*Rhodobaca barguzinensis*	DSM19920	M	ectRABCask-ect	o	betAB>proXWV	/betT/ 2x	o	o	W2	W3	o
Rhodobacteraceae	*Rhodobaculum claviforme*	GOR B7-4	M	ectRABCask-ect	o	o	/betT/ 2x	o	o	o	o	o
Rhodobacteraceae	*Rhodovulum adriaticum*	DSM2781	M	ectRABCask-ect	o	o	/betT/	o	o	W2	o	o
Rhodobacteraceae	*Rhodovulum imhoffii*	DSM 18064	M	ectRABCask-ect	o	o	/betT/ 2x	o	o	o	o	o
Rhodobacteraceae	*Rhodovulum sulfidophilum*	IM 196	M	ectRABCask-ect	o	o	/betT/	o	o	W2	o	o
Rhodobacteraceae	*Rhodovulum sulfidophilum*	DSM 2351	M	ectRABCask-ect	o	o	/betT/	o	o	W2	o	o
Rhodobacteraceae	*Rhodovulum sulfidophilum*	DSM 1374	M	ectRABCask-ect	o	o	/betT/	o	o	W2	o	o
Rhodobacteraceae	*Roseisalinus antarcticus*	DSM 11466	M	ectR-X-ectABCask-ect	o	betABNCIproXWV/betAT	/betT/ 2x	o	o	o	W3	o
Rhodobacteraceae	*Roseivivax isoporae*	LMG 25204	M/H	ectRABCask-ect	o	betABC /<bet>proWX	/betT/ 4x	o	o	o		o
Rhodobacteraceae	*Roseivivax halotolerans*	JCM 10271	M/H	ectRABCask-ect	o	betABC /betI>proWV	/betT/ 3x	o	o	W2		o
Rhodobacteraceae	*Roseivivax halodurans*	JCM 10272	M/H	ectRABCask-ect	fusedMT-MAT	betABTC /betAT/bet>proWX	/betT/ 3x	o	o	W2		o
Rhodobacteraceae	*Roseivivax roseus*	DSM 23042	H	ectRABCask-ect	fusedMT-MTHFR-MS-MAT-SAHase	betABCIproX/proWV	/betT/ 5x	o	W1	o		W4
Rhodobacteraceae	*Rhodosalinus sediminis*	KCTC52478	H	ectRABCask-ect	fusedMT- MS-MAT	betABIproXWV	betT/	o	o	W2	W3	o
Rhodobacteraceae	*Roseovarius nitratireducens*	KTCT52967	M/H	ectRABCask-ect	fusedMT-MTHFR-MS-MAT-SAHase	betACINproXWV	betT/	o	o	W2	W3	W4
Rhodobacteraceae	*Roseovarius halotolerans*	DSM 29507	M/H	ectRABCask-ect	o	betABCINproXWV	/betT/ 2x	o	W1	o	W3	W4
Rhodobacteraceae	*Roseovarius mucosus*	SMR3	M	ectRABCask-ect	o	betACIproXWV	/betT/ 2x	o	o	o	W3	W4
Rhodobacteraceae	*Roseovarius litoreus*	DSM 28249	M	ectRABCask-ect	o	betABCINproXWV	/betT/ 2x	o	o		W3	W4
**Rhodospirillales**												
Acetobacteraceae	*Acidiphilium cryptum*	JF-5	F	ectRABCDask_ect	o	proXWVbetBA	o	o	o	o	W3	o
Acetobacteraceae	*Acidiphilium multivorum*	AIU301	F	ectRABCDask_ect	o	proXWVbetBA	o	o	o	o	W3	o
Acetobacteraceae	*Paracraurococcus ruber*	DSM 15382	F	o	o	o	o	o	o	W2	o	o
Acetobacteraceae	*Rhodopila globiformis*	DSM 161	F	o	o	o	o	o	o	o	o	o
Rhodospirillaceae	*Pararhodospirillum photometricum*	DSM 122	F	o	o	o	o	o	o	o	o	o
Rhodospirillaceae	*Phaeospirillum fulvum*	MGU-K5	F	o	o	o	o	o	o	o	o	o
Rhodospirillaceae	*Phaeospirillum molischianum*	DSM 120	F	o	o	o	o	o	o	o	o	o
Rhodospirillaceae	*Rhodospirillum rubrum*	DSM 1068	F	o	o	proXbetAB/betI/	o	o	o	o	o	o
Rhodospirillaceae	*Rhodospirillum rubrum* 220	DSM 107	F	o	o	proXbetAB/betI/	o	o	o	o	o	o
Rhodospirillaceae	*Rhodospirillum rubrum*	ATCC 11170	F	o	o	o	o	o	W1	o	o	o
Rhodospirillaceae	*Rhodospirillum rubrum*	FR1Mutante-IV	F	o	o	o	o	o	W1	o	o	o
Rhodospirillaceae	*Rhodovibrio salinarum*	DSM 9154	H	ectBC/2 x ectA	GMT-B-DMT-MAT-SAHase	betABIproX	betTproX	proVWopuAC		o	o	W4
Rhodospirillaceae	*Rhodovibrio sodomensis*	DSM 9895	H	ectBC/2 x ectA	GMT-B-DMT-MAT-SAHase	betABIproX	/betT/ 6x	o	W1	o	o	W4
Rhodospirillaceae	*Caenispirillum salinarum*	AK4	M	ectABCD/A/D	fusedMT-MAT	betABIproXWV	/betT/	opuD	W1	o	W3	o
Rhodospirillaceae	*Rhodospira trueperi*	ATCC 700224	M	ectABC	fusedMT-MAT	o	/betT/ 2x	o	W1	W2	o	o
Rhodospirillaceae	*Roseospira marina*	DSM 15113	M	ectABC	MAT-fusedMT	betIBAproX	/betT/ 2x	o	W1	W2	o	o
Rhodospirillaceae	*Roseospira navarrensis*	DSM 15114	M	ectABC	MAT-fusedMT	betIBAproX	/betT/	o	W1	o	o	W4
Rhodospirillaceae	*Roseospirillum parvum*	DSM 12498	M	ectRABC	MAT-fusedMT	o	/betT/ 2x	o	W1	W2	o	o
**Rhodothalassiales**												
Rhodothalassiaceae	*Rhodothalassium salexigens*	IM 261	H	o	GMT-DMT	o	/betT/ 3x	o	W1	o	o	o
Rhodothalassiaceae	*Rhodothalassium salexigens*	IM 265	H	o	GMT-DMT	o	/betT/ 3x	o	W1	o	o	o
Rhodothalassiaceae	*Rhodothalassium salexigens*	DSM 2132	H	o	GMT-DMT	o	/betT/ 3x	o	o	o	o	o
												
**Sphingomonadales**												
Erythrobacteraceae	*Erythrobacter litoralis*	DSM 8509	M	o	GMT-DMT	o		o	o	o	o	o

^a^ see footnote ^a^ in Table 1; Color shades indicate different types of gene and gene associations, also marine (blue) and halophilic (rose) growth response of the bacteria. Color shades indicate different types of gene and gene associations: ectoine synthesis in rose, betaine synthesis from glycine in blue and from choline in green (in association with proU-W3 in pink; transport with betT (light-lila), opuA and D (shades of beige-brown), proU-W1 and proU-W2 shades of green, proU-W3 pink, proU-W4, yellow; also marine (blue) and halophilic (rose-pink) growth response of the bacteria.

**Table 3:** Genes and gene clusters of betaine and ectoine biosynthesis as well as relevant transport systems of phototrophic *Gammaproteobacteria* ^a,b^.

Gene repertoire of Phototrophic Gammaproteobacteria for Osmotic Adaptation									
Family	Species	Strain	Salt	Ectoine	Betaine biosynthesis		Osmolyte transport		
			response	biosynthesis	from glycine	from choline			
				ectABC	GMT-DMT	betAB	betT	opuA	proW1
								opuD	
									
**Cellvibrionales**									
Halieaceae	*Congregibacter litoralis*	KT71	M	o	o	no genes of osmotic stress synthesis and transport			
									
**Chromatiales**									
Chromatiaceae	*Allochromatium vinosum*	MT86	F	o	o	o	o	o	o
Chromatiaceae	*Allochromatium vinosum*	DSM 180	F	o	o	o	o	o	o
Chromatiaceae	*Allochromatium warmingii*	DSM 173	F	o	o	o	o	o	o
Chromatiaceae	*Chromatium okenii* 6010	DSM 169	F	o	o	o	o	o	o
Chromatiaceae	*Chromatium weissei* IM 5910	DSM 5161	F	o	o	o	o	o	o
Chromatiaceae	*Lamprocystis purpurea*	DSM 4197	F	o	o	o	o	o	o
Chromatiaceae	*Thiocystis minor*	DSM 178	F	o	o	o	o	o	o
Chromatiaceae	*Thiocystis violacea*	DSM 207	F	o	o	o	o	o	o
Chromatiaceae	*Thiocystis violascens*	DSM 198	F	o	GMTtruncated,DMT	o	o	o	o
Chromatiaceae	*Thiocapsa imhoffii*	DSM 21303	F	o	o	o	betT/	o	o
Chromatiaceae	*Thiocapsa roseopersicina*	DSM 217	F	o	o	o	betT/	o	o
Chromatiaceae	*Thiocapsa marina* 5811	DSM 5653	M	o	GMT-DMT-betT	o		opuAA,AB,AC-N-GMT,DMT,betT	o
Chromatiaceae	*Marichromatium gracile* 5210	DSM 203	M	o	GMT-DMT	betBAT	betT/ 2x	opuAA,AB,AC-N-opuAC-NN-betT	o
Chromatiaceae	*Marichromatium purpuratum* 984	DSM 1591	M	o	GMT-DMT	o	betT/	opuAA,AB,AC,N,AC,AC,NN,betT	o
Chromatiaceae	*Thiocystis violacea*	DSM 208	M	o	GMT-DMT	o	betTopuAC	opuAA,AB,AC	o
Chromatiaceae	*Imhoffiella purpurea* AK35	AK35	M	o	GMT-DMT	o	betTopuAC	opuAA,AB,AC	o
Chromatiaceae	*Thiorhodococcus drewsii*	AZ1	M	o	GMT-DMT	betBAT/betAB	betT-opuAC/betT	opuAA,AB,AC	o
Chromatiaceae	*Thiorhodococcus mannitoliphagus*	DSM 18266	M	o	GMT-DMT	o	betT-opuAC/betT	opuAA,AB,AC	o
Chromatiaceae	*Thiorhodococcus minor*	DSM 11518	M	o	GMT-DMT	o	betT/ 4x	o	W1
Chromatiaceae	*Thioflavicoccus mobilis* 8321	ATCC700959	M	o	GMT-DMT /GMT-B	o	betT/ 2x	o	W1-N-betT
Chromatiaceae	*Thiococcus pfennigii* 4252	DSM 228	M	o	GMT-DMT	o	betT/ 2x	o	W1
Chromatiaceae	*Thiococcus pfennigii* 4254	Pfennig 8320	M	o	GMT-DMT	o	betT/ 3x	o	W1
Chromatiaceae	*Thiorhodovibrio winogradskyi*	06511	M	o	GMT-DMT	o	betT/ 3x	proVW1-opuAC	
Chromatiaceae	*Rhabdochromatium marinum*	DSM 5261	M	o	GMT-DMT /GMT-B	o	betT/ 3x	proVW1-opuAC	
Chromatiaceae	*Lamprobacter modestohalophilus*	DSM 25653	M	o	GMT-DMT	o	betT/ 3x	o	W1
Chromatiaceae	*Halochromatium roseum*	DSM 18859	M	o	GMT-DMT	o	betT/ 2x	o	W1
Chromatiaceae	*Halochromatium glycolicum*	DSM 11080	H	o	GMT-DMT /GMT-B	o	betT/ 4x	W1-NN-betTopuAC	
Chromatiaceae	*Halochromatium salexigens* IM6310	DSM 4395	H	o	GMT-DMT /GMT-B	o	betT/ 2x	W1-N-betTopuAC-NN-betT	
Chromatiaceae	*Thiohalocapsa halophila* IM4270	DSM 6210	H	o	GMT-DMT	o	betT/ 5x	o	W1
Ectothiorhodospiraceae	*Ectothiorhodospira mobilis*	DSM 237	M	o	GMT-DMT-MAT /GMT-B	o	betT/	betT-opuAA,AB,AC	o
Ectothiorhodospiraceae	*Ectothiorhodospira marismortui*	DSM 4180T	M/H	o	GMT-DMT-MAT /GMT-B	o	betT/	betT-opuAA,AB,AC	o
Ectothiorhodospiraceae	*Ectothiorhodospira marina*	DSM 241T	M/H	o	GMT-DMT-MAT /GMT-B	betABIproX	betT	betT-opuAA,AB,AC	o
Ectothiorhodospiraceae	*Ectothiorhodospira haloalkaliphila*	ATCC 51935	M/H	o	GMT-DMT-MAT /GMT-B	betABIproX	betT/ 2x	betT-opuAA,AB,AC	OpuD
Ectothiorhodospiraceae	*Ectothiorhodospira* sp.	BSL-9	M	o	GMT-DMT-MAT /GMT-B	betABIproX	betT/	betT-opuAA,AB,AC	o
Ectothiorhodospiraceae	*Ectothiorhodospira shaposhnikovii*	DSM 243	M	ectB/	GMT-DMT-MAT	betABIproX		betT-opuAA,AB,AC	o
Ectothiorhodospiraceae	*Ectothiorhodospira magna* B7-7	DSM 22250	M	o	o	o		betT-opuAA,AB,AC	o
Ectothiorhodospiraceae	*Ectothiorhodosinus mongolicus* M9	DSM 15479	M	o	o	o	betT/	o	W1
Ectothiorhodospiraceae	*Thiorhodospira sibirica*	ATCC 700588	M	o	o	o	o	o	o
Ectothiorhodospiraceae	*Halorhodospira abdelmalekii*	DSM 2110	H	ectAB/C	GMT-DMT-MAT-SAHase-MTHFR	o	see proU	opuD/ 2x	W1-betT
Ectothiorhodospiraceae	*Halorhodospira halochloris*	DSM 1059	H	ectABC	GMT-DMT-MAT-SAHase-MTHFR	o	betT/ 2x	opuD	o
Ectothiorhodospiraceae	*Halorhodospira halophila*	IM 9626	H	ectABC	GMT-DMT-MAT-SAHase-MTHFR/GMT-B	o	betT/ 5x	opuD	W1
Ectothiorhodospiraceae	*Halorhodospira halophila* SL1	DSM 244	H	ectABC	GMT-DMT-MAT-SAHase-MTHFR/GMT-B	o	betT/ 4x	opuD	W1-N-betT
Ectothiorhodospiraceae	*Halorhodospira halophila* D	IM 9620	H	ectABC	GMT-DMT-MAT-SAHase-MTHFR/GMT-B	o	betT/ 5x	opuD	W1-N-betT
Ectothiorhodospiraceae	*Halorhodospira halophila* 51/3	IM 9630	H	ectABC	GMT-DMT-MAT-SAHase-MTHFR/GMT-B	o	betT/ 5x	opuD	W1
Ectothiorhodospiraceae	*Halorhodospira neutriphila*	DSM 15116	H	ectABC	GMT-DMT-MAT-SAHase-MTHFR/GMT-B	o	betT/ 3x	o	W1

^a^ footnote as ^a^ in Table 1; ^b^ proW2, proW3, and proW4 are absent from Gammaproteobacteria of this study; Color shades indicate different types of gene and gene associations: ectoine synthesis (rose), betaine synthesis from glycine (blue) and from choline (green); transport with betT (light-lila), opuA and D (shades of beige-brown), proU-W1 (green); also marine (blue) and halophilic (rose-pink) growth response of the bacteria.

**Table 4:** Ectoine and betaine biosynthesis of selected chemotrophic bacteria ^a^.

Family	Species	Strain	Ectoine	Betaine	biosynthesis
			biosynthesis	from glycine	from choline
			ectABC	GMT-DMT	betAB
**Actinobacteria**					
Actinopolysporaceae	*Actinopolyspora halophila*	DSM 43834	ectABC	fusedMT-MAT-o-MS-SAHase-MTHFR	o
Gordoniaceae	*Gordonia alkanivorans*	NBRC16433	ectABC	o	betABT
Mycobacteriaceae	*Mycolicibacterium thermoresistibile*	DSM 44167	ectABCD	o	betA/betB
Streptomycetaceae	*Streptomyces clavuligerus*	ATCC 27064	ectABCD/AB	o	betAB/betI/betT
					
					
**Bacilli/Bacillales**					
Bacillaceae	*Halobacillus halophilus*	DSM 2266	ectABC/D	o	betABbetIopuAC
					
**Proteobacteria/Alphaproteobacteria**					
Rhodobacteraceae	*Jannaschia seosinensis*	CECT7799	ectRectABCask-ect	o	proVWXbetICBA
Rhodobacteraceae	*Maritimibacter alkaliphilus*	HTCC2654	ectRectABCask-ect	o	proVWooXbetICBA
Rhodobacteraceae	*Paracoccus halophilus*	JCM 14014	ectRectABCask-ect	o	proVWXbetIBA
Rhodobacteraceae	*Salipiger bermudensis*	HTCC2601	ectRectABCask-ect	o	proVWXbetICBA
Rhodobacteraceae	*Thioclava pacifica*	DSM 10166	ectRectABCask-ect	o	proVWoXbetIBA
Rhodospirillaceae	*Ferruginivarius sediminum*	WD2A32	ectRectABC/B/C/D	o	proXbetIBA
					
**Proteobacteria/Betaproteobacteria**					
Alcaligenaceae	*Achromobacter xylosoxidans*	SOLR10	ectRABCD	o	betA/betB
Alcaligenaceae	*Bordetella avium*	197N	ectRABCD	o	betA/
Burkholderiaceae	*Paucimonas limoigeni*	DSM 7445	ectRABCD	o	betA/
Oxalobacteraceae	*Herminiimonas arsenicooxidans*	DSM 17148	ectRABCD	o	betA/
					
**Proteobacteria/Gammaproteobacteria**					
Halomonadaceae	*Halomonas elongata*	DSM 2581	ectABC/D	o	proXbetIBA
Halomonadaceae	*Chromohalobacter salexigens*	DSM 3043	ectABC/D	o	proXbetIBA
Halomonadaceae	*Chromohalobacter marismortui*	DSM 6770	ectABC/D	o	proXbetIBA
Haliaceae	*Haliea salexigens*	DSM 19537	ectRectABCask-ect	o	betA/
Chromatiaceae	*Nitrosococcus oceani*	ATCC 19707	ectRCask-ect/ectABD	o	betA/
Chromatiaceae	*Nitrosococcus halophilus*	Nc4	ectRCask-ect/ectABD	o	o
Hahellaceae	*Hahella chejuensis*	KCTC2396	ectABCD	o	betIBAproXWV
Oceanospirillaceae	*Marinomonas mediterranea*	MMB1	ectABC/R/ask-ect	o	betAB / betIproXWV
Ectothiorhodospiraceae	*Nitrococcus mobilis*	NB-231	ectABC	GMT-DMT	betA/
Chromatiaceae	*Acidihalobacter prosperus*	DSM 14174	ectABC	o	o

^a^ Gene clusters are given and “/” denotes a separate locus of the genes in the genome. Color shades indicate different types of gene and gene associations, also marine (blue) and halophilic (rose) growth response of the bacteria.

## Data Availability

Data on gene and genome sequences and accession numbers are contained within supplementary material of the article. All sequence data are available in a publicly accessible repository of gene bank.

## Supplementary Materials

The Supplementary Material for this article is available online at https://www.mdpi.com/2076-2607/9/1/46/s1.
